# A conciliation mechanism for self-organizing dynamic small groups

**DOI:** 10.1186/s40064-016-2516-7

**Published:** 2016-06-21

**Authors:** Minglun Ren, Zhongfeng Hu, Hemant Jain

**Affiliations:** Key Laboratory of Process Optimization and Intelligent Decision-making, Ministry of Education, School of Management, Hefei University of Technology, Hefei, 230009 China; Department of Management, The University of Tennessee Chattanooga, 615 McCallie Avenue, Chattanooga, TN 37403 USA

**Keywords:** Group decision, Self-organizing dynamic group, Collective requirement, Virtual organization, Social network, Internet of things

## Abstract

A group of individuals, organizations or things in internet of things (IoT) often dynamically self-organizes in small groups to accomplish certain tasks. This is common in virtual organization, social networks and the evolving field of IoT. These small groups have different behavioral characteristics than large groups. Members individually have some requirements and contribute some resources to the group. The organization and operation of such a group requires dynamic identification of group requirements that can be fulfilled by available resources and is approved by the group. We apply design science methods to develop an artifact that helps in conciliation of collective requirements and resources of small groups while maintaining each member’s satisfaction. The mechanism also supports dynamic conciliation as members leave and new members join the group. Each member’s requirement is specified as an explicit/implicit objective that is feasible/not feasible based on resources available to the group and whether the requirement is in alignment with other members’ objectives. We validate the artifact by using it for a manufacturing service group and simulating the change in collective group requirements and resources as group membership changes dynamically.

## Background

In modern business, social and technical environments, increasingly a group of individuals, organizations or things [specifically in internet of things (IoT)] dynamically comes together in a self-organizing small group to complete a task and/or accomplish certain objectives. The motivation for organizing a group may be diverse, e.g. a group of vendors may organize themselves in a virtual enterprise to take advantage of new business opportunities or a group of friends may come together to organize a trip or a group of objects connected by IoT devices may dynamically come together to complete a task. The group may be organized opportunistically in an ad-hoc manner and can change dynamically as new members join or current members leave the group. In this environment each member of the group has their own goals and objectives and is willing to contribute certain resources to the group. The group needs to conciliate each individual’s goals and objectives to arrive at common group requirements that can be satisfied with their collective resources. The following examples illustrate the formation of such dynamic groups in various domains and the issues faced by the groups.

A group of small manufacturers creates a dynamic alliance as a virtual organization (Crispim et al. 2015) to take advantage of an emerging market opportunity. Each member contributes its core competencies, skills and resources to the virtual organization. Each member has an equal right to put forward his/her ideas of market requirements and ways to satisfy them. The alliance needs to compare all ideas, consider available group resources and choose the most profitable idea. When a new enterprise joins the alliance with a new market requirement, for example green variants of the base product, the requirements of the alliance change because the alliance needs to manufacture not only the base product, but also its green variant. In this case, the optimal choice of the alliance requirements needs to consider new resources and profit levels for the new product mix.

In a manufacturing plant with IoTs (Zhang et al. 2015), smart things such as operators (human), machines, pallets and materials embedded with sensors exchange information and their current status through wireless networks. As each entity has its own objectives and capabilities to do different tasks, the collection of such dynamic smart entities must find the most preferred set of objectives that can be accomplished with the available resources. Any change in the member entity’s situation or its status needs to be automatically reflected in the feasibility of the corresponding tasks. Based on the priority assigned by the group, new tasks need to be selected based on feasibility, delivery time and resource availability.

Social networks are increasingly being used to bring together individuals or organizations for specific purposes such as completing certain tasks (Wanyuan and Yichuan 2014). For example, when a group is organized for learning or to pursue a certain interest, each member shares the knowledge of his own culture, language and environment. This knowledge can be deemed as a resource to fulfill the group’s needs or interests (Fan et al. 2015). In this case, the group must conciliate its members’ needs and resources to fulfill the group’s objective. The requirements of conciliation of individual objectives to arrive at a group’s requirements based on resources available are same in all three cases.

The problem of determining the collective requirements/objectives of a self-organizing dynamic group that is relatively small is significantly different from the problem of determining mass requirements (the requirements of very large groups such as tweeter user groups), which has been addressed in the literature (Kim et al. 2010). This is because the members of small groups have more intimate relationships; they communicate, interact, and cooperate more closely than large groups (Arrow et al. 2000; Carton and Cummings 2012). Gao and Krogstie (2015) found that actors involved in a dynamic environment need much more cooperation to create value for the group as a whole than for each individual group member. Additionally, in small group applications like IoTs, there is a need to arrive at collective requirements in real time. Previous research has primarily focused on requirements of large groups and has focused on requirement identification based on demographics, such as gender (Fletschner and Carter 2008), income (Constantin et al. 2012; Ghafoor et al. 2010 and Wadud et al. 2009), or age (Sandfeld and Jensen 2005). Some research also takes into consideration environmental factors to explain the transition from implicit requirements to explicit ones (Cysneiros and Sampaio Do Prado Leite 2004; Wang and Zeng 2009; Boman et al. 2012; Kazmierczak and Bogusz-Czerniewicz 2012). The impact of group discussion on choices made by the group has also been explored. However, in small dynamic groups, there are more micro-level interactions and information sharing among group members which affects the development and maintenance of member’s judgment (Gigone 1997; JaB et al. 2008; Ye et al. 2012).

Since self-organizing dynamic groups are often opportunistically organized, there is a need for a systematic approach that can dynamically determine the collective requirements of the group (Guo et al. 2013; Mitra and Poellabauer 2012). We not only need to identify the group’s needs accurately, but also respond to those needs adequately (Florez-Lopez and Ramon-Jeronimo 2012). The members of the group organized to accomplish a task need to cluster their requirements as well as allocate their resources for the mission. Thus, it is important to develop an automated approach to conciliate individual requirements into group requirements that can be fulfilled by the resources available to the group.

In this paper, based on the small group theory and requirements in various domains, we develop an approach for determining collective requirements of small groups and ways to meet those collective requirements based on the resources that can be deployed. We also address the issue of dynamic change in a group’s configuration and their impact on requirements and the feasibility of satisfying the requirements. The mechanism for conciliating requirements of members of a self-organizing dynamic group was developed using a design science methodology widely used in MIS literature (Gill and Hevner 2013; Gregor and Hevner 2013a, b; Von Alan et al. 2004). We deeply analyzed the dynamic relationships between the requirements and the resources of group members. As we focus on the identification of collective requirements, we try to identify the unique requirement and consider the clustering effect as the configuration of the group changes dynamically. All the members of the group contribute information which helps in forming new objectives and defining tasks. The group members share resources for fulfilling the objectives and completing the tasks. We use a state transition approach to simulate the process of conciliating tasks with available resources when members change. The collective requirements are then determined by the tasks that have enough resources and satisfy the group members’ preferences. The approach developed in the paper was validated with the help of an experimental example.

The remainder of the paper is organized as follows: “Literature review and theoretical background” section presents the literature review and theoretical background on small groups and group requirements. “Design science methodology and artifact design” section presents the design science research methodology and development of conciliation mechanism for collective requirements (artifact) based on this methodology. It also develops an approach to select the best requirements for the group by incorporating the preferences and resources as decision factors. “Effectiveness of the proposed conciliation mechanism” section describes an experimental example used to verify the effectiveness of the proposed method and presents the results of research. “Discussion and implications” section discusses the findings and its implications for managers and researchers. Finally, we conclude the paper by summarizing its contributions and directions for future work in “Conclusions, limitations and future work” section.

## Literature review and theoretical background

Requirements have been widely researched in disciplines such as economics (Ingram 2003; Reich 1997), marketing (Moore and Pareek 2009; Pareek 2006), psychology (Coon and Mitterer 2012; Murray 1938) and software engineering (Jayatilleke and Lai 2013; Sutcliffe and Sawyer 2013). Different terms have been used for similar concepts in different fields, such as need being a state of tension that can stimulate us to seek contentment (E Atwater and Duffy 1994); when payment capacity is considered, wants are converted to demands (Moore and Pareek 2009); demand is a desire supported by purchasing power (Kotler 1997); or a requirement is a condition or capability needed by a user (human or system) to solve a problem or achieve an objective (Ebert 2005).

Self-organizing groups have been recognized and studied in various forms, such as autonomous groups in socio-technical systems, enablers of organizational theories, agents of knowledge management, and as examples of complex-adaptive systems (Hoda et al. 2013). In the self-organized groups, group membership is decided by the group members (Li et al. 2014). Additionally, group members must have a common focus, mutual trust, respect, and the ability to reorganize repeatedly to meet new challenges (Jim and Alistair 2001). Minimum critical specification, requisite variety, redundancy of functions and learning to learn are regarded as the four principles of self-organization in a holographic organization (Morgan 1998). Furthermore, Conradt et al. (2009) indicated that individual members of self-organizing groups can increase their influence on group dynamics by changing their behavior, and group movements are led according to the needs of self-organizing groups (Conradt et al. 2009). Petruzzi et al. (2013) proposed self-organizing flexible demand in smart grids, where consumers can cooperate with each other by using their own social capital to get a certain amount of electricity for a certain period of time (Petruzzi et al. 2013). Briscoe and De Wilde (2009) extended physical complexity to provide a greater understanding of multi-agent systems with evolutionary dynamics and investigated the self-organizing aspects of digital ecosystems (Briscoe and De Wilde 2009). Self-organization can be generated in multi-agent systems in several ways: direct interactions between agents using basic principles such as localization, indirect interactions between agents, reinforcement of agent behaviors, cooperation between individual agents, and generic architectures or meta-models (Ye et al. 2012). Obidallah et al. (2014) proposed a multilayered procedural framework for the virtual organizations in order to meet the internal and external requirements of the competitive and rapidly changing environment in which they operate (Obidallah et al. 2014).

Although researchers have provided different understandings of requirement based on their own research areas, we can find the following common elements of such group requirements: requirement is perceptible; it is a state (internal/external, physical/virtual) of being short of something that is perceived by an entity through information acquisition. But not all short-of-something states result in requirement. There must be an equilibrium process during which those states can be satisfied. Requirements contain objectives and require resources the entity might have, such as money and problem solving capabilities. The subject-object relationship is unified in an individual requirement. As heterogeneity is exhibited in individuals’ characteristics, there should be cooperation among different individual entities if they want to fulfill their common objectives while maintaining their satisfaction.

Group requirements have been widely explored to help identify factors that affect group decision- making. Three major perspectives have been used: individual interaction and information sharing that affect group objectives; resource allocation to satisfy group objectives; and preferences that affect the determination of group requirements. Current research in the area of basic determinants that play an important role in the formation of collective requirements of the dynamic self-organizing group is summarized in this section.

### Exchange of requirement information and collective requirement awareness

Marketing literature has identified customers as a heterogeneous group of individuals who differ in their personalities, values, and a range of other characteristics resulting in differing needs (Wang and Tseng 2014). It has been found that groups of individuals generally fail to fully share their information and needs, resulting in a suboptimal choice of alternatives (Lightle et al. 2009). Sharing of useful information expands alternatives, clarifies choice and enables a group to achieve desired outcomes (Mcnie 2007). Groups generally bring together individuals with unique perspectives and information. If pooled together efficiently, groups should be able to achieve superior outcomes.

Groups are often expected to promote cross-fertilization, resulting in better decisions because of their access to a broader range of information, which is due to the unique knowledge distributed among group members (Hollenbeck et al. 1995). It has been shown that groups can make better quality decisions if different members pool available information together (Brodbeck et al. 2007; Dennis 1996). Tai et al. (2012) show that if the initiator of a purchasing group shares information about products and special deals more frequently, members tend to gain a better understanding and are more willing to engage in group buying (Tai et al. 2012). Notwithstanding the benefits of information sharing among members of established groups to make high-quality decisions and to foster creativity and innovation, information exchange between newcomers and existing members of a dynamic group can also increase choices and improve decision quality. Thomas-Hunt et al. (2003) show that socially connected members give greater emphasis to the unique knowledge of socially isolated members than they do to the knowledge of socially connected individuals (Thomas-Hunt et al. 2003).

It can be concluded that when individuals are self-organized together in the collective environment, they exchange information and expand their awareness and this motivates new objectives. Information exchange among members of a self-organizing dynamic group is a process of perceiving the objectives of each member. *Conciliating group members’ objectives to form preferred group objectives is a crucial problem in organizing and managing self*-*organizing dynamic groups.*

### Feasibility of group requirements and resource allocation

Demand is a desire supported by purchasing power (Kotler 1997). Customers’ abilities to pay should be considered when considering requirements for products and services (Reich 1997). A group’s propensity to adopt a product, in general, is driven by a variety of economic factors such as product price, availability, and income (Cojocaru et al. 2013). The resource-based view suggests that the rationale for alliances is the value-creation potential of the firms’ resources that are pooled together. Resource characteristics, such as imperfect mobility, limitability, and substitutability promise accentuated value-creation and facilitate alliance formation (Das and Teng 2000). A resource-based approach can explain strategic alliance formation through expansion and diversification of resource usage, imitation of resources and disposal of resources (Tsang 1998). The collection of unique resources and capabilities that are valuable, rare, inimitable and non-substitutable can provide the alliance a sustainable competitive advantage (Laosirihongthong 2014). Thus, the resource plays an important role in the feasibility of the alliance and its requirements. In general, resource allocation is the essential foundation for filling the gap between the desired state and the actual state of the group. The group requirements will not be feasible if enough resources to fulfill the requirements are not available to the group. Thus, identifying the feasible requirements based on the resources available to the group is an important problem to be addressed.

### Influence of preferences on collective requirement determination

Demand for a product which determines an enterprise strategy is often influenced by customer preferences (Fornell 1992). Customers exhibit heterogeneity in their preferences and buying behavior relative to the same product (Linoff and Berry 2002). Significant work has been done to increase the group consensus level while maintaining consistency at the individual level (Ben-Arieh and Zhifeng 2006; Chen et al. 2015; Chen and Lee 2012). MacDonald et al. developed a framework for understanding preference inconsistencies based on behavior psychology (MacDonald et al. 2009). Li et al. introduced the concept of combinatorial coalition formation for multi-item group-buying with heterogeneous customer preferences to benefit all buyers (Li et al. 2010). Alti et al. (2015) proposed a cloud semantic-based dynamic multimodal adaptation platform to identify situations, inference constraints and determine the necessary adaptations to help achieve different user’s preferences under multiples device constraints and multiple interacting modalities (Alti et al. 2015). In self-organizing dynamic groups the preferences of group members on feasible group requirements varies, which in turn influences the determination of collective requirements. Therefore, to maximize consensus in the group, the preferences of each member should be considered in ranking the alternatives that agree with the majority.

Thus, a self-organizing dynamic group must conciliate its members in three determinants: their objectives, resources and preferences. This is the prerequisite for a self-organizing dynamic group. Therefore, for unfolding the formation mechanism of collective group requirements, three major tasks are identified: (1) identify the three determinants, (2) analyze the way these determinants influence the collective requirements of the group when its members change, and (3) determine the most preferable feasible requirements of the group.

Though the conciliation process potentially has wider scope, when applied to various organizational levels, the determination of information requirements is generally complex, involving a number of different stakeholders, which requires several political, sense-making, and communicative processes (Davidson 2002; Davis 1982). It is difficult to clearly list all objectives, resources and preferences of an organization or individual member. This hinders the automatic information exchange and collaboration of members forming a self-organizing group with adequate interaction.

In the context of IoT, event processing, pattern detection, data mining, and context-aware computing are all important (Wren and Tapia 2006). Information to identify important events needs to be identified. Ganz et al. defined two levels of information abstraction for human/machine interpretable representation of sensor data. They developed domain-independent approaches for processing the large volumes of heterogeneous data in various application scenarios (Ganz et al. 2015). In this paper we focus on IoT, social network and virtual organizations, where the functions of each member entity are predefined and their three determinants can be clearly analyzed. *We assume that a self*-*organizing dynamic group requires each member to be treated as an independent entity and their interrelationships analyzed separately.* This is different from group theories in behavioral and economic disciplines, which emphasize the common behavior of group members.

Based on the above discussion this paper focuses on self-organizing, dynamic small groups and addresses following problems:

- Conciliating group members’ objectives to form preferred group objectives
- Identify the feasible group requirements based on the resources available to the group
- Treat each member as independent entity to separately analyze their interrelationships to help deal with dynamic nature of group.

## Design science methodology and artifact design

In this paper we adopt the design-science research paradigm to create and evaluate an artifact (tool) that can automatically conciliate each group members’ requirements to arrive at common requirements that can be satisfied with the resources available to the group. Additionally we develop a mechanism to dynamically change group’s requirements as group membership changes. The goal of the design-science research paradigm is “to extend the boundaries of human and organizational capabilities by creating new and innovative artifacts” (Hevner et al. 2004, p. 75). In their framework for design-science research, Hevner et al. (2004) specify a set of guidelines. We follow the suggested pattern for presentation of these guidelines as provided by Gregor and Hevner (2013a, b). The first guideline is that design-science research should result in an artifact, which could be a construct, model, method, or instantiation. The artifact described in this paper is a model and methodology. The second guideline relates to problem relevance. That is, the artifact developed should be relevant to the practitioner community. As described above, the problem addressed here is relevant to many domains. The third guideline focuses on design evaluation. We present the evaluation of the method by an experimental example. The fourth guideline is that design-science research must clearly articulate the research contributions. We identify the research contributions of the proposed mechanism in “Conclusions, limitations and future work” section of the paper.

### The conciliation mechanism for collective requirements

Self-organizing dynamic groups are organized with certain goals. Each member of the group has its own personality and preferences; each contributes information or knowledge to the group and provides resources to the group. Thus, the collective requirements of a self-organizing dynamic group have a complex structure. Below we formally define individual and collective requirements in such groups and develop a mechanism for automatically deriving collective requirements.

#### Individual requirements

Goals of an individual member in joining the group relate to the three determinants: objectives, willingness to share resources, and preferences. Objectives of an individual member can be explicit or implicit. In the case of explicit objectives the individual member is aware of objectives through their own information, while implicit objectives are activated by information obtained from other members. Resources are necessary to help achieve the individual members’ objectives and represent their willingness to share these resources. Preferences of individual members imply that they have a greater interest or desire for fulfilling certain objectives than others. Thus, if an objective is preferred and enough resources are available to achieve it, then it is a feasible requirement for the individual member.

#### Collective requirements (CR)

Collective requirements are a set of objectives that are agreed to by the members of the group as group objectives for which they are willing to share their resources. Collective requirements have the same three determinants as individual requirements. Below, we describe a mechanism for deriving them for the group.

Objectives in collective requirements: As a collection of its members’ individual objectives, the objectives in collective requirements represent the awareness of the group to achieve a set of objectives denoted as $CRO$. The group’s awareness of $CRO$ is determined by the information shared by different members of the group. Therefore, for a group with $m (m \ge 2)$ members, if $cro$ represents the individual awareness of a collective requirement, then $CRO$ can be calculated as:

1 $$CRO = \mathop \sum \limits_{i = 1}^{m} cro_{i}$$

This perceived collective requirement gets stored in the group’s knowledge base and remains unchanged for a long period, which leads to a special property of $CRO$. According to this property, $CRO$ changes uni-directionally, which means the degree of a group’s awareness of the requirement can only increase or stay the same. This is because the new objectives of the group can only be formed through information exchange between new and existing members of the group, so the group’s awareness of a requirement will increase when new members join the group. However when a member depart the group their awareness cannot be taken away so it will have no influence on the $CRO$. Zack explored the short term knowledge strategy of organizations, and found firms tend to exploit internal or external knowledge rather than develop new knowledge (Zack 1999), so the awareness of requirements in a self-organizing dynamic group with current knowledge repositories can only increase.

Resources for collective requirements: Resources for collective requirements, denoted as $CRR$, are a collection of members’ resources $crr$ available for accomplishing the objectives in the collective requirements. The resources can be a specific thing, a condition or a one-of-a-kind capability. The $CRR$ can be calculated as:

2 $$CRR = \mathop \sum \limits_{i = 1}^{m} crr_{i}$$

Since $CRR$ the is sum of resources belonging to members, it differs from the $CRO$. The $CRR$ can increase or decrease in types and quantity when the group changes. In particular, when a new member joins the group, $CRR$ will increase, and it will decrease when a member leaves the group. Since many types of resources are required for satisfying the collective requirement, the feasibility of a requirement may change as members leave or join the group. For simplicity of illustrating the effect of resource variation on the requirement feasibility, it is assumed that only one type of resource is needed for a requirement realization.

Preference for collective requirements: Even if the expressed requirements are feasible for the group, all of the requirements may not be realized. The intuitive idea of reflecting on the varying objectives of the group members to accomplish the mission of the group is to consider the preferences of group members as a crucial decision criterion. Thus, arriving at group preference for collective requirements is a mechanism that can be used to satisfy all the group members, denoted as $CRP$.

To achieve maximum consensus in the group, individuals’ preferences for collective requirements are used to make a ranked list of requirements. Each member decides its own preferences for a collective requirement and assigns a weight to each collective requirement. Therefore, if there are *m* members and $n (n \ge 1)$ collective requirements, then the matrix of group preferences can be expressed as:

$$CRP = \left[ {\begin{array}{*{20}c} {crp_{11} } & \cdots & {crp_{1n} } \\ \vdots & {crp_{ij} } & \vdots \\ {crp_{m1} } & \cdots & {crp_{mn} } \\ \end{array} } \right],$$

where, $crp_{ij}$ is the preference of the *i*th member for the *j*th collective requirement (i.e. $CR_{j}$). Let $W = [w_{1} , \ldots ,w_{i} , \ldots ,w_{m} ]$ represent the weights assigned to each member of the group. The weight of each member can be derived based on the member’s contribution of resources and awareness. The group preference for requirements can be calculated by using the following formula (illustrated for the *j*th collective requirement):

3 $$CRP_{j} = \mathop \sum \limits_{i = 1}^{m} w_{i} *crp_{ij} \left( {0 \le w_{i} , crp_{ij} \le 1, \mathop \sum \limits_{i = 1}^{m} w_{i} = 1, \mathop \sum \limits_{i = 1}^{n} crp_{ij} = 1} \right)$$

The final collective requirement to be realized (denoted as CR_F_) can be selected from the ordered list of preferred requirements based on resource availability. Thus, *Collective requirement* can be defined as the most preferred objectives that can be satisfied by the group’s resources.

The collective requirements have self-organizing characteristics. Group members must conciliate themselves with their objective, resources and preferences to obtain a common acceptable objective as the collective requirement. When a new member joins the group with its explicit objectives shared with other group members, some hidden requirements (implicit objectives of other members) of the group will be revealed. Additionally, previously unfeasible collective requirements may become feasible when a new member joins and brings new resources to the group. In contrast, feasible requirements can become unfeasible when a member leaves the group, taking away some necessary resources. As objectives from new members are introduced, member’s preferences may also change, requiring conciliation to be done again. Thus, determination of collective requirements is a dynamic and complex process with members joining or leaving the group.

#### Conciliating collective requirements

A requirement is feasible only if there are sufficient resources available for task fulfillment. Sharing of information and resource between members plays a critical role in conciliating collective requirements. Asymmetric information and/or uneven distribution of limited resources may cause requirements to become unfeasible. For example while a requirement may be unfeasible at a particular time because of lack of sufficient resources, the same requirement may become feasible when information about the availability of certain resources is shared with the group. The sharing of information and resources among the group members might activate implicit objectives of certain individual members and make feasible a requirement that could not be realized by an individual. This process will generate collective requirements that are feasible for the group. In order to represent variant feasibility of a requirement for the group based on the interaction among group members (information and resource sharing), the four states of requirement and the state transition of requirements are defined here.

#### Four states of requirement feasibility

**Fig. 1:**
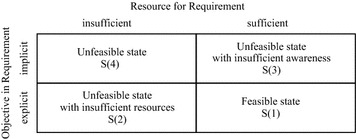
Four states of requirement feasibility

When there is lack of interaction among group members, certain objectives can be explicit for one individual while implicit for others due to information asymmetry. Also, the feasibility of a task may be different for each member based on their own resources. In order to get an insight into the dynamic aspect of requirement feasibility when group members interact with each other, four states of requirement feasibility are defined (Fig. 1).

- *Feasible State S(1)*: When an objective is defined with sufficient information and enough resources to achieve the objective are available, then the requirement is in ‘feasible state’.
- *Unfeasible State with Insufficient Resources S(2):* For an explicit objective of a member, if the desired outcome cannot be achieved due to unavailability of necessary resources, then the requirement is in ‘unfeasible state with insufficient resources’.
- *Unfeasible State with Insufficient Awareness S(3):* If an objective is implicit for a member, the requirement is definitely unfeasible no matter how many resources are available. In this condition the requirement is in ‘unfeasible state with insufficient awareness’.
- *Unfeasible State S(4):* For an implicit objective that the member is unware of, when the entity also falls short of necessary resources needed for requirement realization, such a requirement is in the ‘unfeasible state’ due to insufficient resources and awareness.

Based on the above, the feasibility of a requirement can be determined. However, for implicit objectives, the members will not be aware of them unless there is information exchange among members. Thus, it is impossible to find out requirements in states S(3) and S(4) a priori. However, for theoretic clarity and better understanding of the influence of individual interactions (e.g. information exchange) on requirements feasibility, we propose that the requirement in state S(3) or S(4) can be confirmed from an outsider’s perspective. Bdased on this, individual objectives and resources can be identified and the state of every requirement can consequently be determined.

#### Requirement state transition

There are many dynamic aspects of a self-organizing group. However, in this paper we focus only on the change in membership aspect (members joining or leaving the group with certain information and resources), which affects the group objective and resources and determine the feasible state of a requirement. To help in conciliating requirements of the group, the notion of feasible states introduced above is extended to state transition. This extension allows us to simulate the individual member interactions within the group for collective requirement identification.

**Fig. 2:**
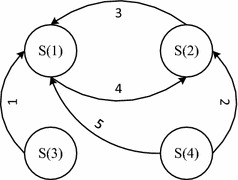
Requirement state transitions for self-organizing dynamic group

As a collection of autonomous entities trying to fulfill their explicit objectives based on their own resources, it is inevitable that individual interactions will vary in terms of information and resources shared, which will influence the feasible state of a requirement. When group members exchange information, some implicit objectives of other members may be activated as they obtain some useful information. It is also possible that some feasible/unfeasible requirements will become un-realized/realized as group members sharing resources. Interaction among group members may create five types of requirement state transition, as illustrated in Fig. 2.

- *Transition 1*: S(3) → S(1). Failure of a group member to fully make use of their own information to discover an implicit objective may occur, although the resources belonging to the group are enough for task accomplishment, which means the requirement may be in state S(3). When information exchange among group members happens, the new information can be utilized by members to make an implicit objective explicit, resulting in the change in state of requirement to S(1).
- *Transition 2*: S(4) → S(2). The requirement is in state S(4) for an individual member due to insufficient resources and awareness. When information exchange among group members happens, the new information can be utilized by members to make an implicit objective explicit, and the requirement state changes to S(2) since there are not enough resources available for the realization of requirements.
- *Transition 3*: S(2) → S(1). For the explicit objective, if the necessary resources become available because one or more members decide to share their resources with the group, then the state of requirement will change from S(2) to S(1), increasing the possibility of group accomplishing its objective.
- *Transition 4*: S(1) → S(2). A requirement can be initially feasible for the group based on available resources. However, it may fall short of resources when the needs of a new member cannot be met, or the available resources decrease to an insufficient level when one or more members leave the group. Then the state of requirement feasibility will change from S(1) to S(2).
- *Transition 5*: S(4) → S(1). If the requirement is initially in state S(4), when the group members interacts by sharing information, the implicit objective can become explicit, and if sufficient resources become available, then the state of requirement feasibility will change to S(1).

CRO can only increase or stay the same, which means that if the implicit objective which the group was unaware of becomes explicit, the requirement will be explicit for a long time with varying degrees of feasibility, so there are no corresponding state transitions in the opposite direction of transitions 1, 2 and 5.

**Fig. 3:**
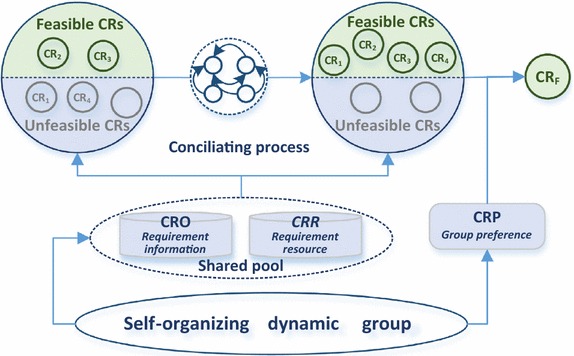
Requirement conciliation process of self-organizing dynamic groups

Based on the above requirement state transitions, we can determine the requirements that can eventually be realized by the group. Furthermore, collective requirements of the self-organizing dynamic group are the requirements that are in the feasible state for each individual member. Then the feasible collective requirements with the maximum satisfaction can be identified by incorporating the group preferences, as shown in Fig. 3.

### A method for identifying collective requirement

Collective requirements transform between the feasible state [S(1)) and unfeasible states (S(2), S(3), S(4)] when group membership changes. S = [S(1),S(2),S(3),S(4)] is defined as the state vector of requirement, the value of state vector element can be 1 or 0, representing whether the requirement is in a particular state or not. Thus the problem of identifying collective requirement CR_F_ can be stated as finding the state vector [1,0,0,0] which best meets the group preferences. Therefore, the best collective requirement $CR_{F}$ for the self-organizing dynamic group can be identified by following 3 steps:

- *Step 1*: Aggregate requirements by pooling individual requirements together. This aggregation generates a list of requirements that the group wants to achieve.
- *Step 2*: Determine the state of each requirement based on available resources and create their state vector.
- *Step 3*: Resolve the preference inconsistency in the group and evaluate the feasibility of each requirement based on member preferences by using function (4), and select the requirement with maximum value based on function (5). This will be collective requirement CR_F_.

4 $$f\left( {CR_{j} } \right) = S_{j} \left( 1 \right)*CRP_{j} = S_{j} \left( 1 \right)*\mathop \sum \limits_{i = 1}^{m} w_{i} *crp_{ij}$$

5 $$f\left( {CR_{F} } \right) = Max\{ f\left( {CR_{1} } \right), \ldots ,f\left( {CR_{j} } \right), \ldots ,f\left( {CR_{n} } \right)\}$$

where $f\left( {CR_{j} } \right)$ evaluates the feasible requirements based on member preferences and CR_F_ selects the requirements with maximum value. “Appendix” describe the details of how states of collective requirements change as members join or leave the group.

### Collective requirement determination by orchestration of group preferences

To determine the feasible requirements that satisfy the majority of the group members (used for ranking candidate choices), preference inconsistency between group members needs to be resolved as group membership changes. Many methods such as “dialog” with the system (Chen and Pu 2004), or an analytical hierarchy process (Saaty 1977) are available for eliciting individual preferences for different items. Let us assume that there are $m^{\prime }$ members in the group after the changes. Then the group preference matrix for the requirements can be derived as:

$$CRR^{\prime} = \left[ {\begin{array}{*{20}c} {crr_{11}^{\prime} } & \cdots & {crr_{1n}^{\prime} } \\ \vdots & {crr_{ij}^{\prime} } & \vdots \\ {crr_{{m^{\prime} 1}}^{\prime} } & \cdots & {crr_{{m^{ \prime} n}}^{ \prime} } \\ \end{array} } \right]\left(\mathop \sum \limits_{i = 1}^{n} crp_{ij}^{\prime} = \mathop \sum \limits_{j = 1}^{{m^{\prime} }} crp_{ij}^{\prime} = 1\right)$$

In order to evaluate each collective requirement in state S(1) using function (4), the weight vector of group members needs to be confirmed. We use the level of individual contribution to the feasibility of collective requirement as criteria to determine the importance of each member. As the feasibility of collective requirements depends on the values of CRO and CRR, we can calculate the importance of each member based on their contribution to CRO and CRR using formula (6) which shows the weight of *i*th group member.

6 $$w_{i} = {{\alpha }}w_{{cro_{i} }} + \beta w_{{crr_{i} }} (0 \le \alpha ,\beta \le 1,\alpha + \beta = 1)$$

where, $\alpha$ and $\beta$ are the contributing coefficients representing the importance of awareness and resources in requirement feasibility. $w_{{cro_{i} }}$ is the contribution of *i*th member to the awareness of collective requirements in state S(1), which can be calculated by using formula (7):

7 $$w_{{cro_{i} }} = \frac{{\mathop \sum \nolimits_{j = 1}^{n} S_{j} (1)cro_{ij} }}{{\mathop \sum \nolimits_{i = 1}^{{m^{ '} }} \mathop \sum \nolimits_{j = 1}^{n} S_{j} (1)cro_{ij} }}$$

In this formula $\mathop \sum \nolimits_{j = 1}^{n} S_{j} (1)cro_{ij}$ is used to evaluate the amount of information shared by the *i*th member that is used for the group awareness of feasible requirements and $\mathop \sum \nolimits_{i = 1}^{{m^{'} }} \mathop \sum \nolimits_{j = 1}^{n} S_{j} (1)cro_{ij}$ represents the gross information content of the group. Similarly, $w_{{crr_{i} }}$, the resource contribution of the *i*th member, can be computed by formula (8):

8 $$w_{{crr_{i} }} = \frac{{\mathop \sum \nolimits_{j = 1}^{n} S_{j} (1)crr_{ij} }}{{\mathop \sum \nolimits_{i = 1}^{{m^{ '} }} \mathop \sum \nolimits_{j = 1}^{n} S_{j} (1)crr_{ij} }}$$

where $\mathop \sum \nolimits_{j = 1}^{n} S_{j} (1)crr_{ij}$ is the quantity of resources belonging to the *i*th member that can be used for the realization of feasible collective requirement j and $\mathop \sum \nolimits_{i = 1}^{{m^{'} }} \mathop \sum \nolimits_{j = 1}^{n} S_{j} (1)crr_{ij}$ is the sum of group resources to be used for all requirements in state S(1).

Based on the above, the weight vector of group ${\text{W}} = [w_{1} , \ldots ,w_{i} , \ldots ,w_{{m^{'} }} ]$ can be calculated. Then, using function (5), feasible requirements based on member preferences can be estimated, based on which the final requirement $CR_{F}$ preferred by the group can be identified.

## Effectiveness of the proposed conciliation mechanism

In this section we present an experimental example to illustrate the feasibility and effectiveness of the proposed conciliation mechanism for collective requirements identification of self-organizing dynamic groups. As cars are generally shrinking in weight, let us consider a self-organizing dynamic manufacturing alliance *A*, which operates in the dynamic manufacturing networks (Papakostas et al. 2013). Alliance *A* can be rapidly configured to produce auto parts from engineered plastic. Alliance *A* is constituted as a loosely coupled self-organizing cooperative dynamic group of diverse partners organized to capitalize on the new opportunities to make plastic automobile fittings, such as lights, dashboard, etc. For simplicity of understanding without losing generalizability, we consider three candidate auto parts $CR_{1}$, $CR_{2}$, and $CR_{3}$. We assume that the minimum resources required for realization of candidate auto parts (requirements) are CRR_1_* = 6, CRR_2_* = 10 and CRR_3_* = 9. The objectives of each alliance member ($E_{1}$, $E_{2}$, $E_{3}$, $E_{4}$) for the requirements and the resources they are willing to share to accomplish the tasks are shown in Table 1.

**Table 1:** Individual CRO and CRR

	Objective in requirement				Resources for requirement		
	$CRO_{1}$	$CRO_{2}$	$CRO_{3}$		$CRR_{1}$	$CRR_{2}$	$CRR_{3}$
$E_{1}$	1	1	0	$E_{1}$	0.86	1.71	3.73
$E_{2}$	1	0	1	$E_{2}$	1.21	3.81	1.78
$E_{3}$	0	1	0	$E_{3}$	1.75	3.11	1.53
$E_{4}$	0	1	0	$E_{4}$	2.15	3.76	2.30
${CRO}_{{j}}$	1	1	1	${CRR}_{{j}}$	5.97	12.39	9.34
$\Delta {CRO}_{{j}}$	0	0	0	$\Delta {CRR}_{{j}}$	−0.03	2.39	0.34

Using formula (9) in “Appendix”, all members’ information can be pooled together to figure out the alliance’s awareness of each requirement. The three requirements are in the wanting list (ΔCRO_j_ = 0). However, based on the resources of *A,* not all three objectives can be realized. Enough resources are available for the realization of $CR_{2}$ and $CR_{3}$. However $CR_{1}$ is short of necessary resources for realization (ΔCRR_1_ = −0.03). Thus, $CR_{1}$ is in state S(2) while $CR_{2}$ and $CR_{3}$ are in state S(1) and the state vector of each requirement is shown in Table 2. In order to find the best requirements that can achieve maximum satisfaction of *A*, profit ratio is used as the preference variable for different requirements (Table 3).

**Table 2:** State of collective requirements of A

	$CR_{1}$	$CR_{2}$	$CR_{3}$
State vector	S(2) [0,1,0,0]	S(1) [1,0,0,0]	S(1) [1,0,0,0]
f(CR_*j*_)	0	0.2512	0.2546

**Table 3:** Profit ratio of different products

	Rate of profit		
	$CR_{1}$	$CR_{2}$	$CR_{3}$
$E_{1}$	0.117	0.076	0.099
$E_{2}$	0.104	0.079	0.083
$E_{3}$	0.091	0.070	0.074
$E_{4}$	0.126	0.062	0.085

If we assume α = β = 0.5, the weight vector of *A* can be computed by using formula (6) w = {0.2252,0.2286,0.2068,0.2394}. Then, the evaluation of each alliance requirement can be determined by function (5) (functional values can also be seen in Table 2). It is obvious that although both $CR_{2}$ and $CR_{3}$ are feasible for *A*, the functional value of $CR_{3}$ is higher than $CR_{2}$, therefore $CR_{F} = CR_{3}$, indicating the best requirement of the alliance.

With highly uncertain market behavior and requirement fluctuations, the alliance chose a short-term cooperation strategy to expand their capacity and their existing production capabilities. To reflect the dynamic nature of the alliance, it is assumed that an enterprise $E_{5}$ joins alliance *A* with a new market opportunity (denoted as $CR_{4}$, and CRR_4_* = 9) which the existing alliance members are not aware of. Table 4 shows the profit ratio for the combination of products in this context. Additionally, after a short period of cooperation with other partners, $E_{3}$ quits the alliance because of its low level of competitiveness. The awareness of requirement $CR_{4}$ and the resource allocation after $E_{5}$ joins the group are illustrated in Table 5.

**Table 4:** Profit ratio of different products for the changed alliance

	Rate of profit								
	$E_{1}$	$E_{2}$	$E_{3}$	$E_{4}$		$CR_{1}$	$CR_{2}$	$CR_{3}$	$CR_{4}$
$CR_{4}$	0.094	0.062	0.052	0.100	$E_{5}$	0.095	0.080	0.081	0.056

**Table 5:** CRO and CRR of E5

	$CR_{1}$	$CR_{2}$	$CR_{3}$	$CR_{4}$
$CRO_{5j}$	0	0	0	1
$CRR_{5j}$	1.09	5.81	1.95	1.87

When $E_{5}$ joins *A* with its unique information and additional resources, $CR_{1}$ changes its state from S(2) to S(1) as the necessary resources for its realization (ΔCRR_1_ = 1.06) become available. The $CR_{4}$ was in state S(2) for $E_{5}$, but it switches to state S(1) for the group when $E_{5}$ joins *A* (ΔCRR_4_ = 1.95). At this time, all four requirements are feasible for *A*. The functional values of each requirement can be calculated using function (5) as (f(CR_1_) = 0.2012, f(CR_2_) = 0.2020, f(CR_3_) = 0.2033, f(CR_4_) = 0.2034). Now $CR_{4}$ is identified as the best requirement for the alliance. However, the state of $CR_{1}$ and $CR_{4}$ will change to S(2) (ΔCRR_1_ = −0.69, ΔCRR_4_ = −1.09) when $E_{3}$ leaves the alliance and takes away some necessary resources. Thus, the evaluation of each requirement needs to be recalculated (f(CR_1_) = f(CR_4_) = 0, f(CR_2_) = 0.2474, f(CR_3_) = 0.2567). After the dynamic changes of alliance members, $CR_{3}$ is again identified as the best requirement for *A*.

**Fig. 4:**
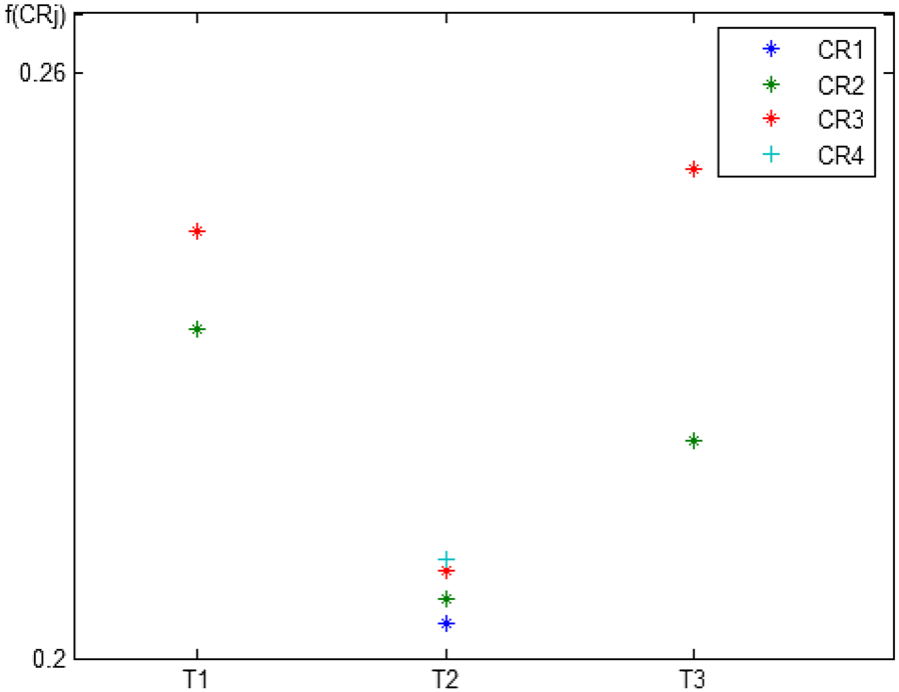
Dynamic evaluations of alliance requirements

Therefore, the collective requirements of the virtual organization (self-organizing dynamic group) dynamically change as members join or leave the organization. Figure 4 shows the state of alliance at different times (T_1_, T_2_, T_3_). Feasibility of requirements change at different times as members join or leave the organization (referred to as state transition). The best requirement can be identified based on the evaluation of all feasible requirements after considering alliance preferences.

## Discussion and implications

With the prevailing use of internet and IoTs, various objects are increasingly being inter-connected. These objects dynamically self-organize themselves into small groups or communities for specific purpose. Members of these small groups have different behavioral traits from those of large groups. Each member in the small group plays a critical role. They must conciliate their resources, objectives and preferences to arrive at collective requirements which can satisfy each member and can be realized by using the resources of the group.

In this research, based on design science methodology we developed the conciliating process for deriving collective requirements of small groups. The conciliating process analyzes group members’ objective, resources and dynamic changes in the group when members join and leave. This allows members to respond to new opportunities, select appropriate members and deal with dynamic changes in the group when member join or leaves. The above small group of entities with common interests can be organized dynamically for specific purpose in cyber space as well as in social life. While the group selects suitable members, the conciliation process presented in this paper can optimize the utilization of resources and can help manage the membership of the dynamically changing group. This approach has broad application in smart-device enhanced industries and can play an important role in the design of complex systems. A few of these examples are provided below.

- Manufacturing and logistics: With smart devices attached to physical entities, smart objects can communicate with one another and with other devices and services over the Internet to accomplish its objectives (Whitmore et al. 2015). This paper’s findings can apply to areas such as manufacturing processes, supply chain logistics and service industries (Flügel and Gehrmann 2008).
- Social network: With increasing interaction of IoT devices with existing social networking services such as Facebook™ and Twitter™ (Vazquez and Lopez-De-Ipina 2008) enhanced services such as providing messages to individuals when they are in proximity of friends, social events of interest, or other activities that may interest them can be offered (Atzori et al. 2016). This allows individuals and smart devices to form special interest groups where each member contributes resources to the group to accomplish common objectives. This research presents a way to automatically find partners from social networks and organize a group in which each member plays an active role.
- Social activities and Virtual organization: are perfect scenarios for the application conciliation mechanism presented in this paper because each individual/organization will share information and resources, and the collective requirement must satisfy every member. Tsang (1998) claimed that the formation of a strategic alliance is based on the diversification and disposal of resource usage (Tsang 1998). Our experiment also shows that the collection of unique resources and capabilities can provide the alliance with new opportunities.

This research has implications from both business and academics perspectives. It provides a better understanding of the conciliation mechanism for self-organizing dynamic group. Based on this understanding our research provides a useful tool for businesses to manage the dynamic nature of groups. From a strategic viewpoint, it is obvious that dealing with changes requires explicit management and attention (Sosa et al. 2004). By documenting the ways of conciliating collective requirements for a self-organizing group, managers can identify key differences (i.e., requirements/objectives, group resource allocation or group preference) between different groups, which can be the foundation of a more general mechanism for a platform for group management.

This research also offers a theoretical extension to group decision making and information systems. The approach presented in this paper can be used to organize various entities, and help business partners to integrate information, resources and activities across organizational and functional boundaries to achieve objectives and optimize decisions. The paper presents a formal process to describe the interaction among members in a small group which can be useful for service management in smart service environment. Thus, our approach provides a structured way for the multi-function members to initiate simultaneous static groups and manage the dynamic process throughout the lifecycle.

## Conclusions, limitations and future work

With advances in computer and networking technology, people and artifacts are linked in cyber-physical space, which results in the formation of diverse groups of entities with dynamic self-organizing characteristics. Members of the group contribute information and resources and share common objectives. Thus, identification of the collective requirements of such groups is crucial. In this paper, we explore how information and resources shared within the group jointly contribute to the collective requirements of the group while considering member preferences. We defined four states of the requirements as feasible state, unfeasible state with insufficient awareness, unfeasible state with insufficient resources and unfeasible state. The state of requirement is determined based on the group’s awareness of the requirement and the resources the group has for the realization of requirements. We analyzed the state transition mechanism which reflects the influence of members joining or leaving the group on the feasibility of the collective requirements. Based on this, we proposed a method to identify the best requirements of self-organizing dynamic groups that consider member preferences.

The mechanism of dynamic group conciliation developed in this paper can help organize a set of autonomous entities to fulfill a mission. The proactive approach of requirement identification of the group provides a practical approach for group requirement analysis. The approach is applicable to a range of service orchestration problems, particularly decision-support in dynamic self-organizing opportunistic groups.

We analyzed influence factors of collective requirements that reveal a conciliating mechanism in the group. Since various group members have different characteristics and personalities, it is a rich area for further exploration. We made some simplifying assumptions to develop the conciliation mechanism for collective requirement identification. Much more work remains to be done. For example we assumed that members would like to share their information and resources with others completely. In many cases this might not be true. There is a causal inter-relationship between share and return, and group members may share information and resources based on some conditions. In this paper, we assume that group objectives are the sum of all members’ objectives, which may be true in an IoT environment. However, in social environments, group members often invoke new requirements through brainstorming. Furthermore, there may be relationships between different resources (e.g. complementary, redundant or duplicate). Also, requirements may not be independent of each other, which will influence the identification process.

## Appendix: State transition of collective requirements

The collective requirement identification is a conciliation process initiated by the requirement state transition triggered by the change in group membership. The state of each requirement needs to be identified based on information pooled from members and available resources. The result of this process is a candidate requirement set used for determination of $CR_{F}$.

### Awareness of requirements

When group members dynamically join the group they bring their unique perspectives, information, and resources. If pooled efficiently, this information can expand alternatives, clarifying choices for the group as well as for individual members. Therefore, to identify states of collective requirements, objectives of group members should be aggregated a priori.

As the information is distributed in an uneven way within the group, objectives can be explicit/implicit for different members. An individual’s awareness of the requirement can be represented as:

$$CRO = \left[ {\begin{array}{*{20}c} {cro_{11} } & \cdots & {cro_{1n} } \\ \vdots & {cro_{ij} } & \vdots \\ {cro_{m1} } & \cdots & {cro_{mn} } \\ \end{array} } \right],$$

where *cro*_*ij*_ denotes the awareness of *j*th requirement by *i*th member of the group. If a member has information for the activation of this objective, then $cro_{ij} = 1$, otherwise $cro_{ij} = 0$. Thus, group awareness of each requirement, for example the *j*th requirement, can be calculated by formula (9):

9 $$CRO_{j} = \mathop \sum \limits_{i = 1}^{m} cro_{ij} = \left\{ {\begin{array}{*{20}c} {1, \mathop \sum \nolimits_{i = 1}^{m} cro_{ij} \ge 1} \\ {0, \mathop \sum \nolimits_{i = 1}^{m} cro_{ij} = 0} \\ \end{array} } \right.$$

If there is no asymmetry in information within the group, then the group becomes aware of an objective if at least one member in the group is aware of it, $\mathop \sum \nolimits_{i = 1}^{m} cro_{ij} \ge 1$, representing an implicit objective becoming an explicit one. However, if there is no information exchanged within the group, then $CRO_{j} = 0$. Based on this, group awareness of each requirement can be determined when different members pool available information together.

### State of collective requirements

Any change in group composition may trigger a state transition for one or more collective requirements, since it may change awareness of the requirements and/or resources available. Thus, to determine a set of feasible requirements, awareness of requirements (described above) and state transition for each requirement needs to be recomputed every time a member joins the group. Similarly, every time a member leaves the group, the state transition of each feasible requirement needs to be recomputed to ensure that the requirement is still feasible. The matrix CRR represents the shared resources available from each member for each requirement:

$$CRR = \left[ {\begin{array}{*{20}c} {crr_{11} } & \cdots & {crr_{1n} } \\ \vdots & {crr_{ij} } & \vdots \\ {crr_{m1} } & \cdots & {crr_{mn} } \\ \end{array} } \right]$$

An element of the matrix $crr_{ij}$ represents the resources belonging to the *i*th member that are used for the realization of the *j*th requirement. The total resources available for *j*th requirement from the entire group can be computed using formula (10).

10 $$CRR_{j} = \mathop \sum \limits_{i = 1}^{m} crr_{ij}$$

Given the value of requirement awareness $CRO_{j}$ and resources available for realizing the requirement $CRR_{j}$, criteria are needed to measure the gap between the feasible state and the actual state of the requirement. The feasible state of requirement awareness and resources are presented as $CRO_{j}^{*}$ and $CRR_{j}^{*}$, where $CRO_{j}^{*} = 1$ represents that enough information about requirement j has been shared for the group to be aware of $CR_{j}$. The minimum level of resources required to realize $CR_{j}$ is represented by $CRR_{j}^{*}$. The requirement’s state can then be evaluated using formulas (11) and (12):

11 $$\Delta CRO_{j} = CRO_{j} - CRO_{j}^{*}$$

12 $$\Delta CRR_{j} = CRR_{j} - CRR_{j}^{*}$$

Once members’ objectives and resources are confirmed, the state vector of each requirement (as $CR_{j}$ illustrated in Table 6) can be computed. The table shows that $CR_{j}$ is a feasible requirement of the group only when $\Delta CRO = 0$ and $\Delta CRR \ge 0$. Furthermore, we can also derive the state vectors of $CR_{j}$, e.g. $S_{{CR_{j} }} = [1,0,0,0]$, representing the state of $CR_{j}$ as S(1).

**Table 6:** State vector of $CR_{j}$

$\Delta CRO_{j}$	$\Delta CRR_{j}$	
	<0	≥0
−1	$S\left( 4 \right):S_{{CR_{j} }} = \left[ {0,0,0,1} \right]$	$S\left( 3 \right):S_{{CR_{j} }} = \left[ {0,0,1,0} \right]$
0	$S\left( 2 \right):S_{{CR_{j} }} = \left[ {0,1,0,0} \right]$	$S\left( 1 \right):S_{{CR_{j} }} = \left[ {1,0,0,0} \right]$

Information asymmetry explains why the state of requirement can change from S(4) to S(2) as well as from S(3) to S(1) through information exchange within the group. However, the free flow of information in the group will make awareness of the requirement stay at the same level even if some member leaves the group. Thus, the value of CRO may increase if a new member joins the group while it will remain unchanged when a member leaves the group. In contrast, the value of CRR changes in a bi-directional way whenever members join or leave the group.
